# Laboratory-Based Prospective Surveillance for Community Outbreaks of *Shigella* spp. in Argentina

**DOI:** 10.1371/journal.pntd.0002521

**Published:** 2013-12-12

**Authors:** María R. Viñas, Ezequiel Tuduri, Alicia Galar, Katherine Yih, Mariana Pichel, John Stelling, Silvina P. Brengi, Anabella Della Gaspera, Claudia van der Ploeg, Susana Bruno, Ariel Rogé, María I. Caffer, Martin Kulldorff, Marcelo Galas, Group MIDAS - Argentina

**Affiliations:** 1 Departamento Bacteriología, Instituto Nacional de Enfermedades Infecciosas ANLIS “Dr C. G. Malbrán”, Buenos Aires, Argentina; 2 Department of Medicine, Brigham and Women's Hospital, World Health Organization Collaborating Centre for Surveillance of Antimicrobial Resistance, Boston, Massachusetts, United States of America; 3 Department of Population Medicine, Harvard Medical School and Harvard Pilgrim Health Care Institute, Boston, Massachusetts, United States of America; 4 Servicio de Antígenos y Antisueros. Instituto Nacional de Producción de Biológicos (INPB) - ANLIS “Dr Carlos G. Malbran”, Buenos Aires, Argentina; The Johns Hopkins University, United States of America

## Abstract

**Background:**

To implement effective control measures, timely outbreak detection is essential. *Shigella* is the most common cause of bacterial diarrhea in Argentina. Highly resistant clones of *Shigella* have emerged, and outbreaks have been recognized in closed settings and in whole communities. We hereby report our experience with an evolving, integrated, laboratory-based, near real-time surveillance system operating in six contiguous provinces of Argentina during April 2009 to March 2012.

**Methodology:**

To detect localized shigellosis outbreaks timely, we used the prospective space-time permutation scan statistic algorithm of SaTScan, embedded in WHONET software. Twenty three laboratories sent updated *Shigella* data on a weekly basis to the National Reference Laboratory. Cluster detection analysis was performed at several taxonomic levels: for all *Shigella* spp., for serotypes within species and for antimicrobial resistance phenotypes within species. *Shigella* isolates associated with statistically significant signals (clusters in time/space with recurrence interval ≥365 days) were subtyped by pulsed field gel electrophoresis (PFGE) using PulseNet protocols.

**Principal Findings:**

In three years of active surveillance, our system detected 32 statistically significant events, 26 of them identified before hospital staff was aware of any unexpected increase in the number of *Shigella* isolates. Twenty-six signals were investigated by PFGE, which confirmed a close relationship among the isolates for 22 events (84.6%). Seven events were investigated epidemiologically, which revealed links among the patients. Seventeen events were found at the resistance profile level. The system detected events of public health importance: infrequent resistance profiles, long-lasting and/or re-emergent clusters and events important for their duration or size, which were reported to local public health authorities.

**Conclusions/Significance:**

The WHONET-SaTScan system may serve as a model for surveillance and can be applied to other pathogens, implemented by other networks, and scaled up to national and international levels for early detection and control of outbreaks.

## Author Summary

Shigellosis causes dysentery and kills an estimated 1.1 million people per year worldwide, 60% of them children under the age of 5. The infectious agent is *Shigella* spp, transmitted from person to person by fecal-oral route or via ingestion of contaminated food or water. Having a system for early detection of outbreaks would be very useful for implementing control measures that help reduce the number of affected patients, economic losses and prevent the dissemination of antimicrobial resistance. We present the application of a space-time permutation scan statistic implemented within the free SaTScan software for laboratory based surveillance of Shigella cases in six provinces from Argentina. SaTScan was applied on the data loaded into WHONET databases (an electronic laboratory data system used world-wide) in the six provinces from April 2009 to March 2012. The project allowed the identification of 32 events, including several of particular public health importance for their duration or number of affected patients. It also strengthened the relationship between the laboratory and epidemiology staff. In conclusion, the combination of WHONET laboratory data and SaTScan analysis can detect important community outbreaks of antimicrobial-resistant shigellosis in a timely manner, to make a difference to public health.

## Introduction

In view of the increasing movement of people, animals, and food products around the globe, new strategies and collaborations are urgently needed to detect the emergence of microbial threats and implement effective control measures. National and regional electronic laboratory-based surveillance collaborations based on routine clinical laboratory test results, as recommended by the WHO-Global Foodborne Infections Network [1] and the WHO-Advisory Group on Integrated Surveillance of Antimicrobial Resistance [2], offer the potential for real-time monitoring of evolving microbial populations. Sophisticated technologies for differentiating among strains and for processing information have proliferated and been incorporated into surveillance [3]. However, advances in organizational aspects such as timeliness of data entry and analysis and integration of local site-of-care laboratories into national and international surveillance networks have developed more slowly [4]. Statistical analysis of laboratory data for the detection of disease outbreaks in the community or in hospitals has also lagged, and existing statistical approaches have tended to focus on temporal trends [5], [6], [7], [8], largely ignoring the geographic component of pathogen population dynamics.

To be most useful for public health purposes, laboratory-based surveillance should 1) be specific, i.e. be capable of distinguishing (a) among species and preferably variants within species and (b) among antimicrobial resistance profiles within those taxonomic groups; 2) have timely electronic data entry; 3) integrate multiple laboratories using uniform protocols and a uniform database; 4) be linked to and used by the agencies responsible for disease control; and 5) implement statistical methods for detecting departures from background levels in both time and space, rather than relying solely on visual inspection of data.

Of the bacterial pathogens causing diarrhea, *Shigella* spp. is one of the most prevalent and most consistently associated with dysentery and persistent diarrhea [9]. Shigellosis kills an estimated 1.1 million people per year worldwide, 60% of them children under the age of 5 [10], and can result in reduced growth in children who survive. *Shigella* species appear highly adaptable to selective pressure and have developed resistance to a number of antimicrobials with patterns of resistance varying temporally and geographically with antimicrobial usage patterns [11], [12], [13], [14]. Highly resistant clones of *Shigella* have emerged in Argentina [15], [16], [17]. Recently, the unique *Shigella flexneri* serotype X variant, which emerged in China in 2001, has rapidly spread, including through Argentina [18], undergoing frequent serotype switching and acquiring resistance to multiple antimicrobials in the process [19].

We report here our experience with an evolving, integrated, laboratory-based, near real-time surveillance system now operating in six contiguous provinces of Argentina, building on a prior retrospective study [20]. It represents real-world surveillance rather than a static system pre-defined in a formal protocol. This system includes all of the desired laboratory-based surveillance system elements listed above and may serve as a model for surveillance not only of *Shigella* spp. but of other community-acquired pathogens in Argentina and elsewhere.

## Materials and Methods

### Surveillance population and period

The microbiology data used for shigellosis prospective surveillance came from a subset of the national Argentine network for monitoring antimicrobial resistance, WHONET- Argentina, which was established in 1986 by the Ministry of Health through the Dr. Carlos G. Malbrán National Institute of Infectious Diseases (INEI). The group is named after WHONET, a free software for the management of microbiology laboratory data developed by the WHO Collaborating Centre for Surveillance of Antimicrobial Resistance promoted by the World Health Organization. Led by the National Reference Laboratory at INEI, WHONET-Argentina currently includes 89 clinical laboratories representing all geographic jurisdictions and captures detailed data on human pathogens and their susceptibility profiles from routine diagnostic specimens.

WHONET-Argentina hospitals were selected to participate in this surveillance initiative on the basis of the completeness and timeliness of data entered into the national WHONET database in previous years, a commitment to send updated data on a weekly basis, and *Shigella* serotyping ability. An additional criterion was the strength of the respective local public health systems in integrating laboratory, epidemiology, food science, and environmental health efforts for the investigation of outbreaks and sources of infection.

On the other hand, surveillance of *Shigella* infections at the national level is currently conducted by provincial hospitals that report the number of cases weekly to the Ministry of Health.

**Figure 1:**
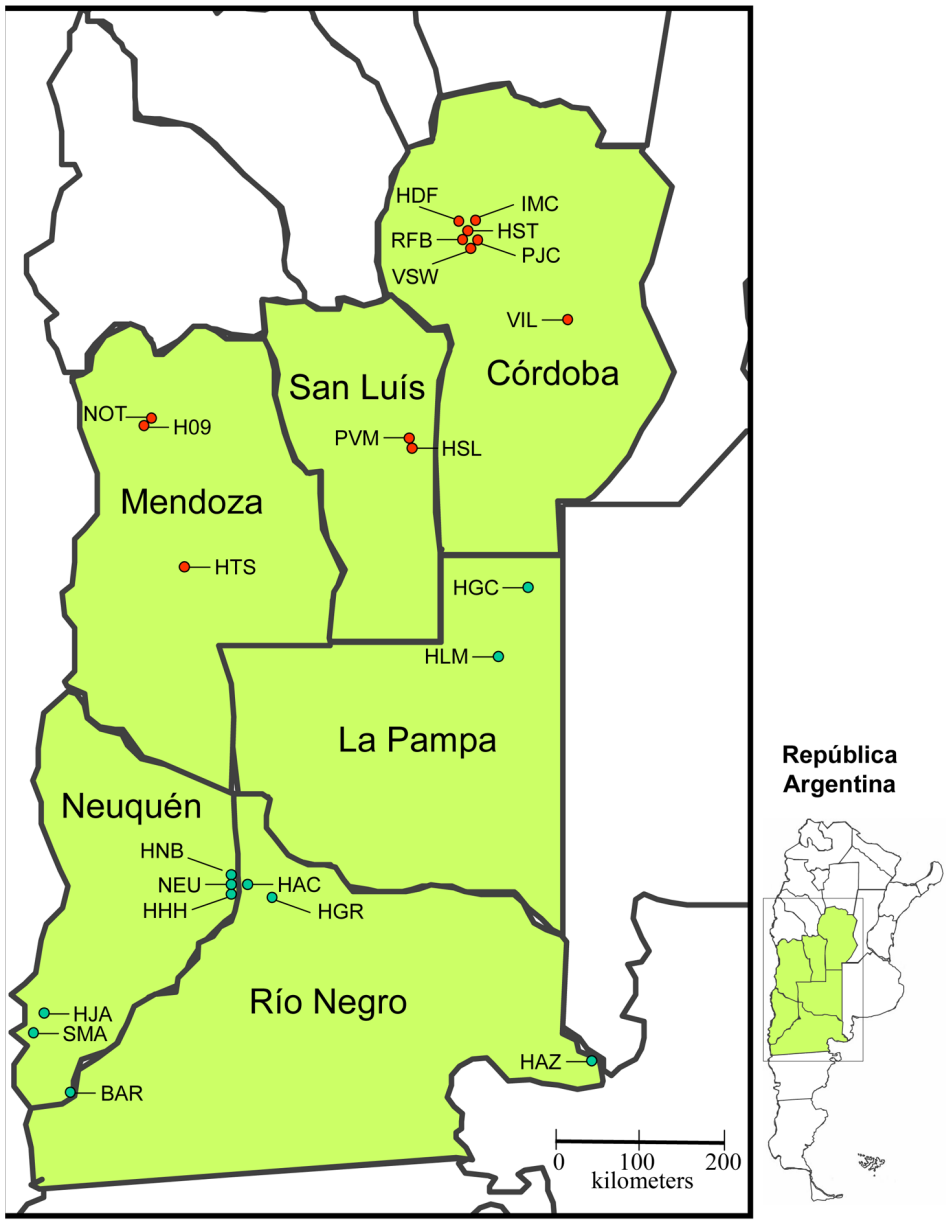
Geographic distribution of laboratories including in this study. The 23 participating laboratories of six provinces of Argentina are indicated with a green circle : 11 hospitals in the three contiguous provinces HAC, BAR, HAZ and HGR from Río Negro province (RN); HLM, HGC from La Pampa province (LP); HHH, NEU, HJA, HNB, SMA from Neuquén province (NEU); and with a red circle: 12 additional hospitals from the three additional contiguous provinces: PJC, RFB, IMC, HST, VIL, HDF and VSW from Córdoba province; PVM and HSL of San Luis province and H09, NOT and HTS from Mendoza province.

The period covered by this report was from April 1, 2009 through March 31, 2012. The number of laboratories and provinces participating increased during this period (Table 1), with growth in laboratories' historical data, capacity for complete and timely data entry, and ability to meet the other inclusion criteria. For the first year, seven hospitals in the three contiguous provinces of La Pampa (LP), Neuquén (NQ), and Río Negro (RN) participated. In March 2010, four satellite clinics in the same three provinces were added. In January 2012, 12 additional laboratories from the three additional contiguous provinces of Córdoba (CBA), Mendoza (MZA), and San Luís (SL) were incorporated (Table 1). Thus, by the end of the evaluation period, 23 laboratories in six provinces were included (Figure 1).

**Table 1:** Settings of the WHONET-SaTScan surveillance system over the April 2009–March 2012.

Date	Laboratories included (laboratory code)	Province (province code)	Changes in WHONET	Changes in SaTScan or its implementation
April 2009	7 labs			SaTScan 7.0.1; max. radius of 110 km.
	Hospital Cipolletti	Río Negro (RN)		only single-lab clusters detectable
	Hospital Zonal Bariloche (BAR)			
	Hospital Lucio Molas (HLM)	La Pampa (LP)		
	Hospital Gobernador Centeno (HGC)			
	Hospital Heller (HHH)	Neuquén (NEU)		
	Hospital Castro Rendon (NEU)			
	Hospital Junín de los Andes (HJA)			
March 2010	4 labs added:			
	Hospital Artimedez Zatti (HAZ)	Rio Negro (RN)		
	Hospital General Roca (HGR)			
	Hospital Centenario Dr Natalio Burd (HNB)	Neuquén (NEU)		
	Hospital San Martín de los Andes (SMA)			
June 2011				CPD resistance profile added
September 2011				SaTScan 9.1.1; max. radius of 200 km,
				single- and multiple- lab clusters detectable
January 2012	12 labs added:		*S. flexneri* AA479 added	
			as specific serotype	
	Hospital Pediátrico del Niño Jesús (PJC)	Córdoba (CBA)		
	Clínica Privada Reina Fabiola (RFB)			
	Hospital Infantil Municipal de Córdoba (IMC)			
	Hospital de Niños de la Santísima Trinidad (HST)			
	Hospital Villa María (VIL)			
	Hospital Regional Domingo Funes (HDF)			
	Clínica Vélez Sarsfield (VSW)			
	Policlínico Regional de Villa Mercedes (PVM)	San Luis (SL)		
	Policlínico Central de San Luis (HSL)			
	Hospital Central de Mendoza (H09)	Mendoza (MZA)		
	Hospital Pediátrico Dr Humberto Notti (NOT)	Córdoba (CBA)		
	Hospital Schestakow (HTS)			
	Hospital Pediátrico del Niño Jesús (PJC)			
	Clínica Privada Reina Fabiola (RFB)			

### Microbiological characterization and data-processing

#### Data collection at the participating laboratories

Stool specimens taken from patients with diarrhea seeking healthcare at participating hospitals and clinics were investigated for the presence of enteric pathogens. Isolation and biochemical identification of *Shigella* spp. were performed using conventional methods [21]. All strains were serotyped using antisera provided by the National Institute of Biological Production (INPB), part of the National Administration of Health Laboratories and Institutes (ANLIS). Susceptibility tests were performed by the disk diffusion method according to Clinical and Laboratory Standards Institute recommendations of 2012 [22]. Susceptibility to the following drugs was evaluated: ampicillin, trimethoprim/sulfamethoxazole, nitrofurantoin, ciprofloxacin, nalidixic acid, cefpodoxime, and fosfomycin, as specified in the annually reviewed Working Protocol of WHONET - Argentina [23].

Microbiological and demographic details of the shigellosis cases were entered into the WHONET 5.6 microbiology database at each participating laboratory. These included patient date of birth, sex, clinical diagnosis, epidemiologic risk factors, co-morbidities, specimen date and type, the bacterial species isolated, serotype, and resistance phenotype for the above-mentioned antimicrobials. A WHONET filter was applied to include only the first sample from each patient in a 60-day period. The latitude and longitude of each participating laboratory and clinic were obtained from Google Earth using the postal address.

#### Data analysis at the central laboratory

Each participating hospital sent its full WHONET database to the National Reference Laboratory at INEI on the Wednesday following the end of each Sunday-to-Saturday epidemiologic week. At INEI, these data were analyzed every Thursday to look for statistical signals suggesting clustering of shigellosis cases in time and space as described in the statistical section below. Signal detection analyses were performed at several taxonomic or genotypic levels: for all *Shigella* spp. aggregated together, for each of the individual species (*S. flexneri*, *S. sonnei*, *S. boydii*, and *S. dysenteriae*), for serotypes within species (*S. flexneri* serotype 1, 2, etc.), and for antimicrobial resistance phenotypes within species. Analyses at higher taxonomic levels can have greater statistical power to find clusters at more specific levels due to incorporating more information about background rates. Even though susceptibility to seven antimicrobials was routinely tested by clinical laboratories, most *Shigella* isolates were susceptible to all agents, with two exceptions: ampicillin (AMP) and trimethoprim/sulfamethoxazole (SXT). Therefore, at the beginning of the collaboration, the focus of analyses was on the four possible phenotypes with these two agents: susceptible to both, resistant to both, resistant to AMP but not SXT, and resistant to SXT but not AMP. In response to the recognition by WHONET-Argentina coordinators of growing resistance to cefpodoxime (CPD), analysis of the eight resistance phenotype combinations with all three agents (AMP, SXT, and CPD) was initiated in June 2011 (Table 1).

During the period of this study, a new serotype of *S. flexneri* was identified in Argentina, recently recognized as a *S. flexneri* serotype X variant [Viñas M, van der-Ploeg C et al., unpublished data]. Initially, participating laboratories entered these isolates into WHONET as atypical *Shigella flexneri* with “fxx” code indicating non-typeable *S. flexneri*. A new code “479” (referring to the antiserum developed by the INPB) was created to permit specific tracking of this new serotype named *S. flexneri* AA479 (Table 1). This code has been used systematically by participating laboratories since January 2012.

#### Molecular strain typing

*Shigella* isolates associated with statistically significant signals identified with the cluster detection method described below were sent from hospital laboratories to INEI and subtyped by pulsed field gel electrophoresis (PFGE) for comparison with each other and with other isolates recorded in the national database (NDB). The PulseNet standardized protocols for *S. sonnei* [24] and *S. flexneri* [25] were applied. The NDB was created by the application of these protocols with the use of a universal standard (*S.* Braenderup) for normatization of the gels in the frame of the PulseNet network. Briefly, genomic DNA embedded in agarose plugs was digested with *Xba*I enzyme for *S. sonnei* and with *Not*I enzyme for *S. flexneri*, and generated fragments were separated by electrophoresis with linear switch times of 2.54 to 54 sec. and 5 to 35 sec for *S. sonnei* and *S. flexneri*, respectively. Genetic relatedness between the isolates that showed indistinguishable PFGE profiles with those enzymes was confirmed with a second enzyme, *Bln*I for *S. sonnei* and *Xba*I for *S. flexneri*. The results were analyzed using BioNumerics software (Applied Maths), and dendrograms were constructed applying the DICE coefficient and UPGMA (Unweighted Pair Group Method with Arithmetic Mean), with a band position tolerance window of 1.5% as established by PulseNet. The criteria for interpretation of PFGE considered the variability of the species under study, the epidemiological context and the frequency of the patterns identified based on information in the national database [26]; also we applied the criteria by Tenover et al. that allowed to assign the isolates to categories of relatedness based on the difference in the number of bands [27].

### Space-time permutation scan statistic

To detect localized shigellosis outbreaks in near real-time, we used the non-parametric space-time permutation scan statistic [28], as was previously used in a pilot study of historical data in the WHONET-Argentina database . The method searches for statistically significant clusters of *Shigella* cases in space and time, using cylindrical scanning windows with a circular base of variable location and radius representing geographical space and a variable height representing the number of days in a potential cluster (ending on the day for which the analysis is being done). It does not require population-at-risk data and makes minimal assumptions about the time, location, or size of outbreaks. The method adjusts for any purely geographical variation in disease incidence, whether due to urban vs. rural, north vs. south, dry vs. humid conditions, etc. There is no need to adjust for any of these variables explicitly. Rather, the adjustment is done non-parametrically, using a permutation-based approach that is conditional on the total area counts summed over all days. Similarly, the method adjusts for any purely *temporal* trends in the data, such as seasonal variation or day-of-week effects. This is done non-parametrically, by day, by using a permutation-based approach that is conditional on the total daily counts summed over all geographic areas. With a seasonal infection such as shigellosis, this ability to automatically adjust for seasonal variation is critical; without such an adjustment it would be difficult to distinguish between epidemiologically important outbreaks and the expected annual increase in shigellosis cases observed each summer. In contrast to other cluster detection methods that adjust for seasonal variation using a parametric model, the space-time permutation scan statistic does not require multiple years of historical data. The paper by Kulldorff et al. [28] describes the method in more detail.

In looking for clusters, we set the maximum temporal cluster length at 30 days, meaning clusters of 1, 2, 3, and up to 30 days' duration could be detected. Because data were updated on a weekly rather than daily basis and because of potential delays in data entry, data transmission, or data availability (e.g. in the ascertainment of organism serotype), prospective analyses were run not only for the last date in the dataset but also for each day in the prior several weeks to ensure that recent clusters were not missed. In each of these day-specific analyses, the prior 365 days of data were used as a historical baseline. From April 2009 to August 2011 we used SaTScan 7.0.1 to search for single hospital clusters; from September 2011 through March 2012, we used SaTScan 9.1.1 to search for single or multi-hospital clusters with a maximum radius of 200 kilometers (Table 1) (Clusters of that apparent or real size could occur if a contaminated food were distributed regionally or if an area of transmission were located between two participating hospitals, leading some patients to go to one hospital and others to the other).

Per analysis run, the statistical inference is adjusted for the multiple testing inherent in the many potential cluster locations, sizes, and time lengths, and is expressed in terms of a recurrence interval [29]. If a detected group of cases is determined to have a recurrence interval of 400 days, for example, this could be an event of epidemiologic significance. But it could be simply due to chance -during any 400-day period, the expected number of signals of that strength or stronger is one, when the null hypothesis of no clusters is true. Thus, the higher the recurrence interval, the less likely that the observed clustering could be attributed to random variation. In this study, we considered a grouping of cases with a recurrence interval of 365 days or longer to be a statistical “signal” and worth communicating (after basic data quality checking) to relevant local or provincial authorities for possible epidemiologic and molecular investigation. Using 365 days as our recurrence interval threshold for notifying public health authorities means that, on average, we could have expected to see one false positive signal per year within each analysis level (genus, species, serotype, and resistance profile). Since statistically significant space-time groupings of cases may be caused by chance or by organizational or procedural factors such as changes in hospital participation, specimen collection practices, or laboratory testing procedures, it is important that statistical signals be evaluated through traditional epidemiologic means before concluding that they are indications of true disease outbreaks.

Calculations for the space-time permutation scan statistic were done using the free SaTScan™ software [30], as imbedded in the free WHONET software.

### Terminology

In the rest of this paper, we use the term “signal” to refer to the detection by SaTScan of a group of cases clustered in space and time with a recurrence interval of 365 days or more. We use the term “event” to refer to a group of signals overlapping in space and time, which may represent a single potential disease outbreak. The term “cluster” is used generically, referring to a group of cases regardless of whether or not these cases are truly related to each other epidemiologically.

### Epidemiologic investigation

When a signal was detected in weekly analyses, a report including data on each patient was sent by INEI analysts to laboratory and epidemiology personnel in the affected hospital and province. These messages were usually sent on Fridays, but later if there were delays in analysis.

The decision by local authorities of whether to investigate a signal depended in part on the recurrence interval and in part on other criteria such as number of patients, location details, and timing and specificity of the signal (homogeneous clusters detected by resistance phenotype or serotype were considered to be more reliable than heterogeneous clusters detected at the *Shigella* genus level). Since the purpose of this real-world surveillance system was to support local public health, no uniform signal investigation protocol was imposed; investigations variously included patient or clinician surveys, additional sampling of either contacts or suspected sources (food or water) for *Shigella* spp., and trace-back of suspected food products.

The events identified by SaTScan confirmed as outbreaks with investigation by local authorities and the confirmation of strains' relatedness by PFGE were analyzed and informed to National Surveillance System.

## Results

There were 32 statistically significant events: 2, 12, 3 and 2 of *S. flexneri* serotypes 1, 2, 3, and AA 479 respectively; 11 of *S. sonnei*; and 2 of *S. boydii* serotype 2 (Table 2 and Table 3). Seventeen of the 32 events were found at the resistance profile level (and some at higher levels also). All 6 participating provinces had events. During the study period, 21 of the events occurred primarily during the Argentine summer months of October–March, while 11 occurred primarily in April–September. The median event duration was 48.5 days (minimum: 3, maximum: 94). The median number of patients in a signal was 21.5 (minimum: 2, maximum: 41).

**Table 2:** Detection of events of *Shigella* by the SaTScan software, April 2009–December 2011 period.

								Surveillance of laboratory (PFGE)			
N° Event	Organism	Analysis Level	Resistance Profile	Prov/Lab	Period (month/day/year)	Max. n° patients	Max. RI	n isolates analyzed by PFGE	n (%) of isolates representing to the MFP in the event typed^a^	n (%) isolates: profiles “related”	n new profile
										n (%) isolates: profiles “not related” to the MFP using a quantitative definition :bd^b^	n existing profile
											n related profile in the NDB
**From analyses including the original three provinces**											
1	*S. flexneri* 2	Resistance	AMP	LP/HLM	03/18/09–04/08/09	9	5000	3	3 (100%)		1 new profile
2	*S. flexneri* 2	Resistance	Susceptible	NQ/NEU	03/18/09–04/12/09	3	1667				
3	*S. flexneri* 2	Resistance	AMP	LP/HGC	07/30/09–08/15/09	4	10000				
4	*S. flexneri* 2	Resistance	AMP-SXT	LP/HGC	08/02/09–08/06/09	3	667				
5	*S. sonnei*	Resistance	SXT	RN/BAR	09/15/09–10/09/09	4	3333	3	2 (67%)		1 new profile
										1 (33%) 1 bd	1 related profile
6	*S. flexneri* 2	Species		LP/HLM	10/19/09–11/06/09	5	1111	4	3 (75%)		1 existing profile
										1 (25%) 1 bd	1 related profile (Event 1)
7	*S. sonnei*	Resistance	SXT	LP/HGC	12/25/09–03/09/10	34	10000	14	7 (50%)		1 new profile
										7 (50%) 1 to 3 bd	5 related profiles
8	*S. flexneri* 2	Resistance	AMP	RN/BAR	01/20/10 only	2	769				
9	*S. flexneri* 2	Resistance	AMP	RN/HAC	02/05/10–03/01/10	9	10000	8	4 (50%)		1 existing profile
										4 (50%) 1 bd	1 related profile
10	*S. sonnei*	Species		LP/HLM	03/23/10–04/26/10	11	3333	7	4 (57%), R: SXT		1 new profile
										2 (28%)≥6 bd, R: AMP-SXT	2 new profiles
										1 (14%)≥6 bd, S: AMP-SXT	1 new profile
11	*S. sonnei*	Species		LP/HLM	05/06/10–06/09/10	13	10000	4	2 (50%), R: AMP-SXT		1 existing profile
										1 (25%) 1 bd	1 related profile
										1 (25%)≥6 bd, R: SXT	1 existing profile (Event 10)
12	*S. sonnei*	Species		RN/HGR	03/08/10–04/12/10	11	10000	9		5 (55%)≥6 bd	7 existing profiles
		Resistance	SXT		03/08/10–04/12/10	9	10000			4 (44%)≥4 to 6 bd	
13	*S. flexneri* 2	Species		RN/HAZ	08/17/10–08/28/10	3	556	2		2 (100%) 2 bd	2 new profiles
		Resistance	AMP		07/26/10–08/27/10	3	588				
14	*S. flexneri* 2	Species		RN/HAZ	11/24/10–11/28/10	6	3333	4	2 (50%) and 2 (50%) 1 bd		2 existing profiles
		Resistance	AMP		11/16/10–11/27/10	6	435				
15	*S. flexneri* 2	Serotype		RN/BAR	09/27/10–10/05/10	2	1000				
16	*S. sonnei*	Species		RN/HAZ	12/19/10–03/23/11	41	10000	15	7 (50%)		1 new profile
		Resistance	AMP - SXT		12/19/10–02/13/11	22	10000			5 (33%) 1 to 2 bd	4 related profiles
										3 (20%) 2 to 3 bd	
17	*S. flexneri* 3	Serotype		NQ/HHH	02/21/11–02/24/11	7	5000	7	7 (100%)		1 new profile
		Resistance	Susceptible		02/21/11–03/01/11	8	10000				
18	*S. boydii* 2	Species		RN/HAC	03/06/11–03/12/11	2	833	2	2 (100%)		1 existing profile
19	*S. sonnei*	Species		LP/HGC	06/13/11–06/20/11	4	625	3	2 (67%)	1 (33%) 4 bd	2 existing profile
20	*S. flexneri* 1	Serotype		NQ/HNB	03/31/11–04/06/11	3	10000	2	2 (100%)		1 existing profile
21	*S. sonnei*	Species		RN/BAR	04/01/11–05/02/11	5	5000	4	3 (75%)		1 new profile
		Resistance	AMP - SXT		03/12/11–05/02/11	3	400			1 (25%) 1 bd	1 related profile (Event 16)
22	*S. flexneri* 3	Serotype		NQ/NEU	08/29/11–09/03/11	2	10000	2	2 (100%)		1 new profile
23	*S. flexneri* 3	Serotype		RN/BAR	09/20/11–09/27/11	2	10000				
24	*S. flexneri* 1	Serotype		NQ/HHH	12/13/11–12/17/11	2	2000	2	2 (100%)		1 existing profile

Prov/Lab: Province/Laboratory.

MFP: (the most frequent pattern).

a : % indistinguishable isolates with the first enzyme and confirmed with the second enzyme.

b : bands of difference (bd).

R: Resistance.

S: Susceptible.

**Table 3:** Detection of events of *Shigella* by the SaTScan software, January 2012–March 2012 period.

								Surveillance of laboratory (PFGE)			
N° Event	Organism	Analysis level	Resistance Profile	Province/Laboratory	Period (month/day/year)	Max. n° patient	Max. RI	n isolate analyzed By PFGE	n (%) of isolates representing to MFP in the event typed^a^	n (%) isolates: profiles “related”	n new profile
										n (%) isolate : profiles “not related” to the MFP using a quantitative definition : bd^b^	n existing profile
											n related profile in the NDB
**From analyses including all six provinces**											
25	*S. flexneri* 2	Serotype		CBA/IMC	01/12/12–02/09/12	19	1000	12	7 (58.3%)		1 existing profile
				+RFB						1 (8%) 1 bd	1 related profile
										4 (33%) 4 to 5 bd	4 existing profiles
26	*S. sonnei*	Species		CBA/IMC	01/03/12–02/17/12	26	1000	18	5 (28%) and 5 (28%) 1 bd		2 existing profiles
				+RFB+HST						1 (5%) 2 bd	1 related profile
										7 (39%) 4 to 5 bd	4 existing profiles
27	*S. flexneri* 2	Serotype		RN/HAZ	03/07/12–03/21/12	4	909	4	4 (100%)		1 existing profile
28	*S. sonnei*	Species		MZA/NOT	12/07/11–02/02/12	24	3452	6		6 (100%)≥6 bd	4 existing profiles
				+HTS							2 related profiles
29	*S. sonnei*	Species		NQ/RN/HAC	02/14/12–03/17/12	9	1244	7	3 (42.8%)		1 new profile
				+HNB+NEU						2 (28%) 1–2 bd	3 related profiles
										2 (28%) 3 bd	
30	*S. boydii*	Resistance	SXT	MZA/NOT	01/26/12–02/23/12	7	3162	9	4 (44.0%)		1 existing profile
		Species			12/28/11–02/09/12	13	833			1 (11%) 1 bd	4 related profiles
										3 (33%) 2 to 3 bd	
										1 (11%) 5 bd	
31	*S. flexneri* 479	Serotype		LP/HLM	02/10/12–03/08/12	4	5211	5	(40%) and 2 (40%) 1 bd		2 existing profiles
		Resistance	AMP - SXT		02/23/12–03/08/12	5	9940			1 (20%) 2 bd	1 related profile
32	*S. flexneri* 479	Resistance	AMP - SXT	NQ/HHH	02/29/12–03/28/12	10	2294	11	9 (81.8%)		1 existing profile
		Serotype			03/24/12–03/28/12	5	1172			2 (18%) 1 to 2 bd	1 related profile

MFP: (the most frequent pattern).

a : % indistinguishable isolates with the first enzyme and confirmed with the second enzyme.

b : bands of difference (bd) to the MFP.

Twenty-six of the 32 events were investigated further by PFGE analysis, which confirmed a close relationship among the isolates (with the first and second enzyme) for 22 (84.6%) of the 26. In contrast, in Events 10, 11, 12, and 28 (all involving *S. sonnei*, PFGE showed diverse genetic subtypes, and we considered these events to be largely chance concentrations of cases, not potential diseases outbreaks. Seven of the events, Events 5, 7, 14, 16, 17, 31, and 32, were investigated epidemiologically, which revealed links among the patients (Table 4), consistent with the PFGE findings for these events.

**Table 4:** Events with epidemiologic evidence.

Event	Organism	Resistance profile	Prov/Lab	Period (month/day/year)	First signal (Fs)	Starting of investigation	n patients with linkage	Nature of linkage	Source identified/confirmed or not	Intervention made
5	*S. sonnei*	SXT	RN/BAR	09/15/09–10/09/09		October 2009	2 of 2	family linkage	No source identified	Hygiene recommendations
										Food handling through local media
14	*S. flexneri* 2		RN/HAZ	11/24/10–11/28/10	11/27/10	Before Fs	14 of 23	family linkage	No source identified	Hygiene recommendations
		AMP		11/16/10–11/27/10	11/27/10					
16	*S. sonnei*		RN/HAZ	12/19/10–03/23/11	12/20/11	February 2011	4 of 6	family	No source identified	Hygiene recommendations
		AMP - SXT		12/19/10–02/13/11	12/20/11			neighborhood linkage	Deficient sanitary conditions	
17	*S. flexneri* 3		NQ/HHH	02/21/11–02/24/11	02/21/11	Before Fs	48 of 80	social linkage -wedding-	No *Shigella* isolates from food	Food control
		Suscep.		02/21/11–03/01/11	02/21/11			patients from	Deficient hygiene conditions	Recommendations on food higieny
								Argentina and Chile	in preparation of foods.	Reporting to Chile via International
										Health Regulation and PulseNet LA
31	*S. flexneri* 479		LP/HLM	03/07/12–03/08/12	03/07/12	March 2012	5 of 5	family linkage	No source identified	Hygiene recommendations
		AMP- SXT		02/23/12–03/08/12	02/29/12			(shared a meal)		
32	*S.S. flexneri* 479		NQ/HHH	03/24/12–03/28/12	03/27/12	March 2012	11 of 11	neighborhood linkage	No source identified	Hygiene recommendations
		AMP- SXT		03/24/12–03/28/12	03/27/12				Deficient sanitary conditions	

Prov/Lab: Province/Laboratory.

LA: Latin America.

Twenty-six of the 28 events considered to represent or possibly represent disease outbreaks were detected before hospital staff was aware of any increase in the number of *Shigella* isolates. The others were Events 14 and 17. In Event 14, *S. flexneri* 2 in Rio Negro, the hospital bacteriologist suspected the outbreak before the appearance of the first WHONET- SaTScan signal, because the majority of isolates were from patients from the same family. Event 17 was a cluster of *S. flexneri* 3 associated with a wedding in Neuquén province, which was attended by 150 people, including some from Chile. More than half the participants became ill, and this outbreak was reported immediately to public health authorities at both national and international levels before the laboratory results could be incorporated into the WHONET database and analyzed.

**Figure 2:**
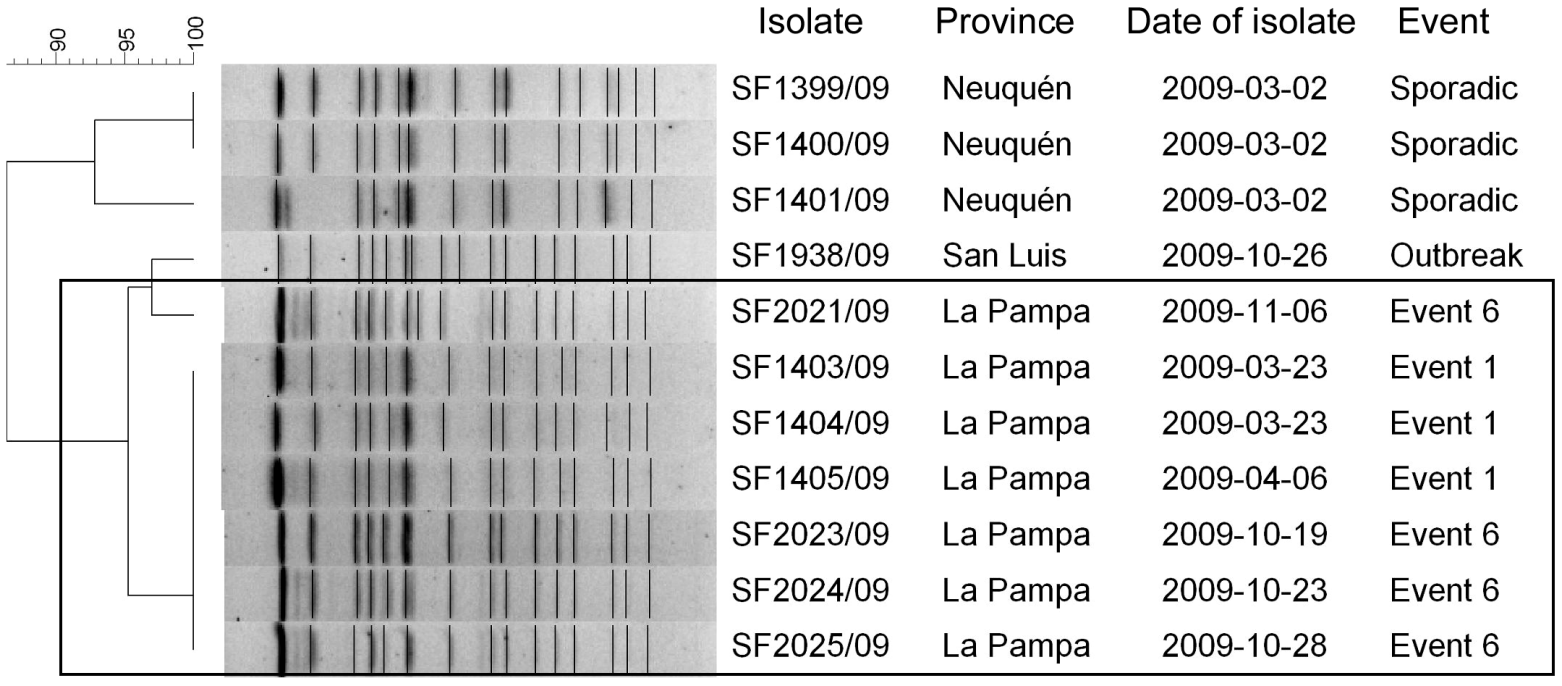
Dendrogram showing the genetic relatedness of isolates of *S. flexneri* 2 included in Event 1 and 6. Isolates recovered in Santa Rosa, La Pampa (Event 1 and 6), and selected isolates for comparison, including one from an outbreak in San Luis and three from sporadic cases in 2009. The rectangle highlights isolates of *S. flexneri* 2 recovered in March–April 2009 (Event 1) and October–November 2009 (Event 6).

The system detected events of public health importance. For example, Events 16 and 21, both in Rio Negro, involved *S. sonnei* resistant to both AMP and SXT, an infrequent resistance profile. A number of long-lasting and/or re-emergent clusters were also detected, represented by 4 pairs of related events: PFGE patterns were very similar for Events 1 and 6, *S. flexneri* 2 in La Pampa (Figure 2), and for Events 13 and 14, *S. flexneri* 2 in Rio Negro. Events in each pair were separated from each other by at least 3 months. Events 10 and 11 of *S. sonnei* in La Pampa were separated by one month and included two persistent patterns and single subtypes circulating simultaneously. Events 16 and 21, *S. sonnei* with the unusual AMP-SXT resistance phenotype, were centered in two cities (Viedma and Bariloche) in Rio Negro province. The predominant PFGE patterns in the latter two events differed by only one band, which is not considered a significant difference in PFGE analysis for *Shigella* when person-to-person transmission is a prominent feature and the outbreak persists in time [26] Event 16 lasted from December 2010 to March 2011, while Event 21 occurred in April to May 2011.

**Figure 3:**
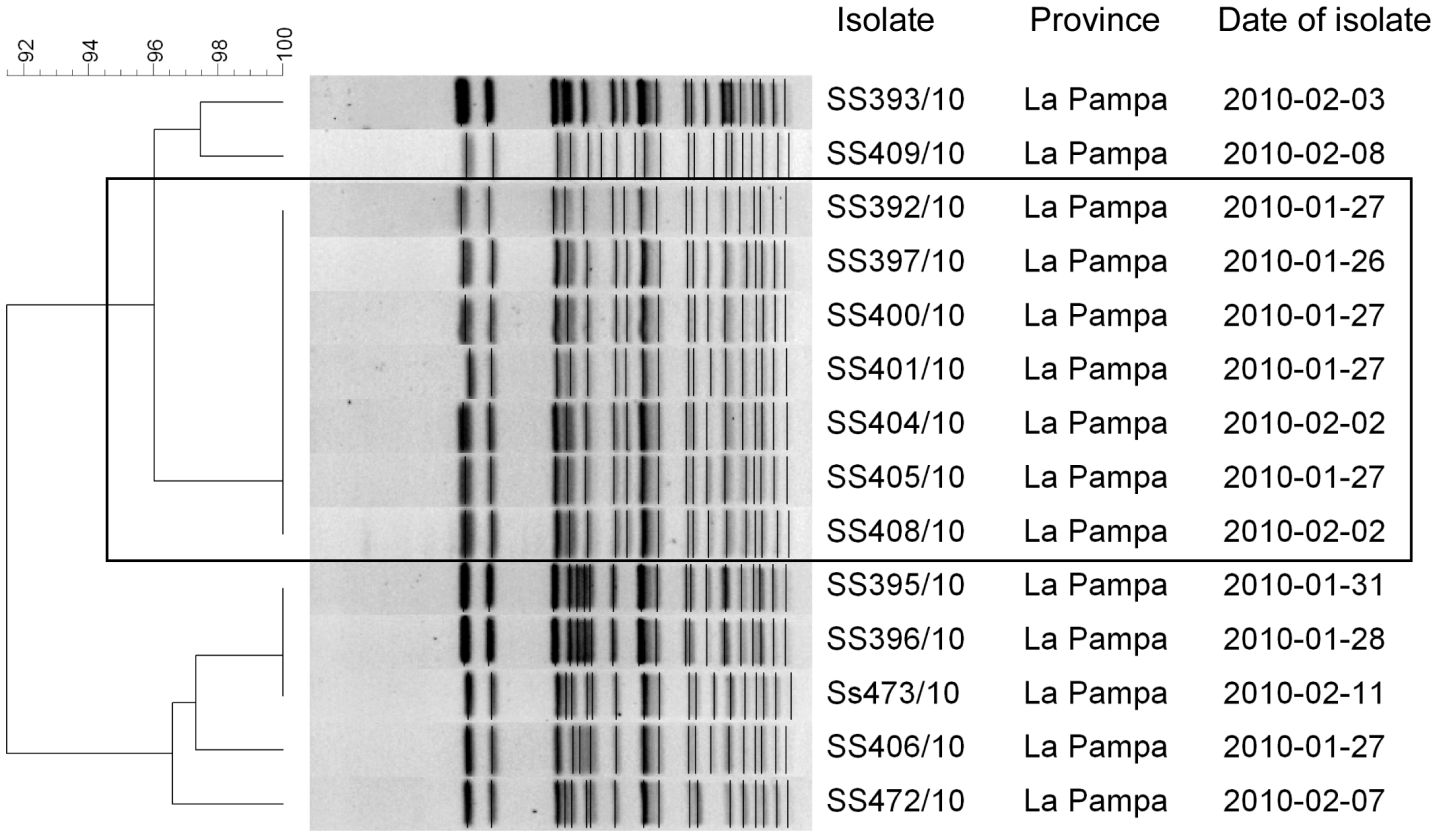
Dendrogram showing the genetic relatedness of *S. sonnei* SXT resistant isolates included in the Event 7. Isolates recovered in Santa Rosa, La Pampa in January – February 2010. The rectangle highlights the 7 *S. sonnei* isolates with an indistinguishable PFGE pattern which had not previously been recorded in the National Data Base (NDB). The remaining 7 isolates identified statistically and epidemiologically as part of Event 7, exhibited high genetic relatedness (from 91.4 to 97.4% similarity, 1 to 3 bands of difference) to the most frequent pattern within the event, confirming the relation of the cases.

**Figure 4:**
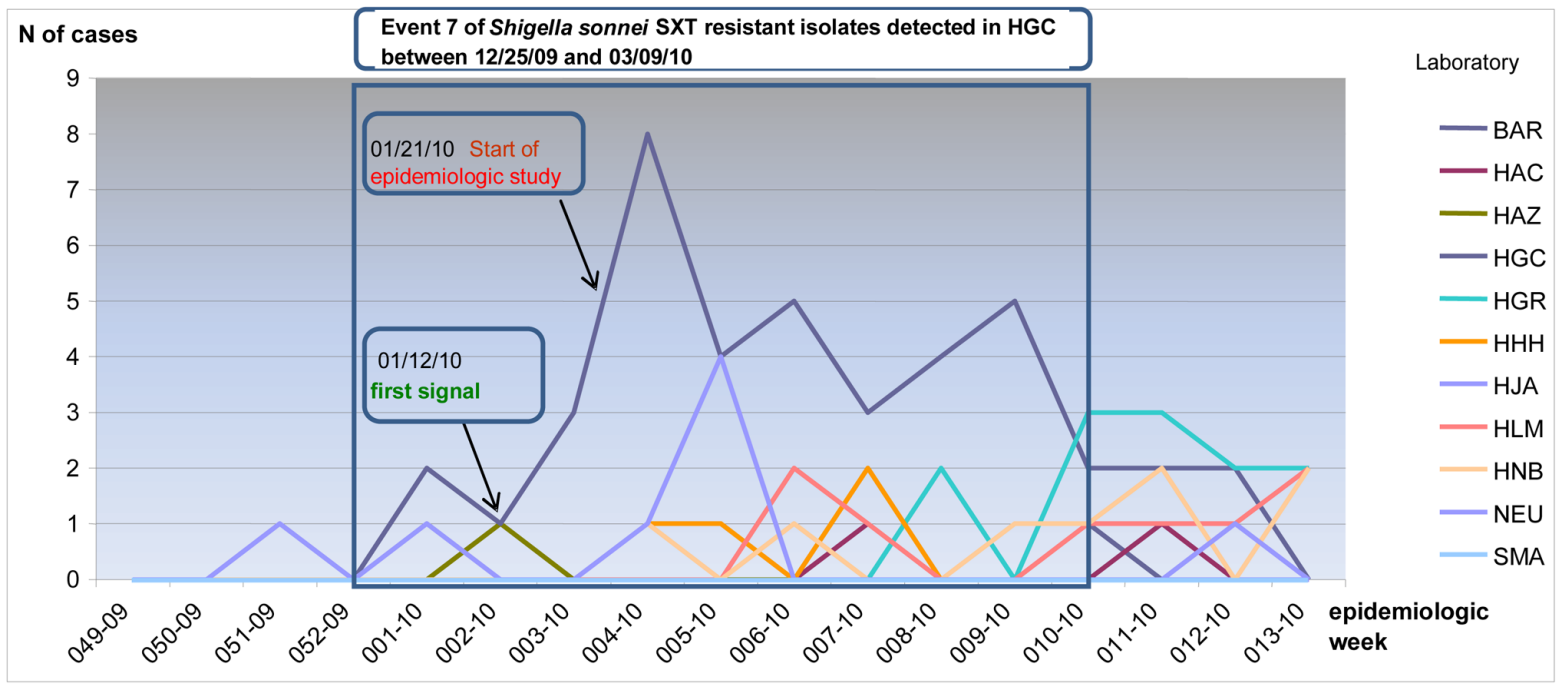
Time series of cases of *Shigella sonnei* isolates detected by SaTScan programme from December 2009 to March 2010. The rectangle highlights the time series of cases corresponding to the detection and evolution of Event 7.

Two other events are worthy of mention due to their duration or size. Event 7, caused by SXT-nonsusceptible *S. sonnei* in La Pampa province, lasted from December 2009 to March 2010, with 34 isolates. Among 23 cases of diarrhoea studied trough clinician surveys, 14 (60.9%) were epidemiologically linked. Seven of 14 SXT-non-susceptible *S. sonnei* isolates analyzed by PFGE shared a new pattern in the national database and the other 7 were closely related to this pattern with similarities between 91.4% to 97.4%, 1 to 3 bands of difference. (Figure 3). No common source was identified, but most of the cases were associated with two neighboring households found to be epidemiologically linked and to have deficient sanitary conditions. Figure 4 shows the time series of cases, for the period from December 2009 to March 2012, to highlight the detection and evolution of event 7. Event 17, the wedding outbreak in Neuquen province, was noteworthy in that it was detected by our surveillance system on the basis of only 7 isolates when in fact the total number ill, according to public health investigation, was closer to 75. The 7 *S. flexneri* 3 isolates from the patients showed indistinguishable PFGE patterns.

In general, no common source could be confirmed in the events, even though food and water samples were analyzed in several instances; this may be due to the difficulty for the isolation of *Shigella* from this kind of sample. Nevertheless, the epidemiologic studies could determine sanitary deficient conditions and probable routes of transmission, mainly from person to person. Furthermore, deficiencies in the conditions for food conservation and elaboration were identified in some events. On this basis, control and prevention measures included recommendations on hygiene and food handling, as well as a notification to the International Health Regulation for an event that affected patients from Chile and Argentina (Table 4).

## Discussion

In three years of active, near real-time surveillance, building on an earlier, purely retrospective pilot study [28], our system detected 32 shigellosis events ( Table 2 and 3 ). Independent suspicion or discovery of only 2 of the 28 events considered suspect of outbreak occurred prior to detection of the first signal by WHONET-SaTScan. Of the 26 events for which we have PFGE evidence, 22 appeared to represent groups of truly genetically interrelated cases, including 9 new patterns (1 of them closely related to pattern identified previously) and 13 subtypes identified before in the NDB , with supporting evidence of epidemiologic linkage for 7.

The 32 detected events represented a broad range of the *Shigella* variants circulating in Argentina and were distributed among the six participating provinces. Some were of particular public health importance because of long duration or number of patients, e.g. Events 7 and 16, or because of a distinct resistance profile, e.g. Events 16 and 21, both *S. sonnei* AMP-SXT. Four pairs of events that were related according to PFGE patterns may have represented additional long-lasting outbreaks.

The two known outbreaks in these six provinces that were not detected by WHONET-SaTScan could not have been found by the system. One, a plasmid-conferred cefpodoxime-resistant cluster of *S. sonnei* in February-March 2011, could not have been detected because cefpodoxime resistance was not one of the phenotypes analyzed at the time. However, it did show up as a non-statistically significant cluster of SXT-AMP-nonsusceptible resistant *S. sonnei*, with RI = 116. The other, of *Shigella flexneri* AA479, appeared in one of the new provinces in August–September 2011, but this was before the new laboratories' data were incorporated and analyzed and before the variant was given a specific code in WHONET.

In Argentina, under the auspices of the international laboratory network PulseNet, it has been possible to maintain active surveillance using PFGE to detect circulating clones [16], [31]. When PFGE results are communicated to local public health agencies, they inform investigation into possible sources of contamination and their persistence over time [32]. PFGE results of the type we often saw one predominant pattern with other closely related genetic subtypes in the same event are common in *Shigella* spp., particularly in long-lasting events with person-to-person transmission, such as Events 7 and 16. Others have reported widespread outbreaks in which this mode of transmission was confirmed by molecular typing results and epidemiologic data [33], [34]. Where person-to-person spread is a prominent feature of the outbreak, more variability is expected [26], [27]. In each of two outbreaks with point-source exposures, event 17 and the outbreak of *S. flexneri* AA479 in the provinces that had not yet been incorporated into the surveillance system, the PFGE patterns were indistinguishable.

There are several limitations to this study. As is often the case in evaluations of surveillance systems, there was no known set of outbreaks that could serve as a gold standard against which to compare all the events. Some events were studied by public health local authorities, while all the cases were reported to the national surveillance system. Therefore, it was not possible to calculate such measures as sensitivity, specificity, or negative predictive value. PFGE testing did stand in as a strong validation method, and 22 (84.6%) of 26 events for which PFGE was done showed evidence of close genetic relatedness. However, close relatedness by itself does not prove that isolates belong to a single outbreak, and accompanying epidemiologic studies were not carried out for every event, so we cannot claim to know the positive predictive value of our system. Also, the system was not pre-specified in a static protocol, having changed in important ways over the three years: the inclusion of additional laboratories, changes in parameter settings, and changes in the kinds/phenotypes of *Shigella* for which clusters could be detected (Table 1). The results may have looked somewhat different had we included all 23 laboratories and the final parameter settings and *Shigella* variants from the start. Finally, we did not compare results of different cluster detection methods applied to the same data. We selected the space-time permutation test because it does not require population-at-risk (denominator) data and it adjusts for purely spatial and purely temporal variation and can do so without multiple years of historical data. It would be worthwhile in a future methods-oriented endeavour to compare the performance of this method with other cluster detection methods.

In large measure, this still-evolving, real-world, laboratory-based surveillance system satisfies criteria for public health utility, including that it 1) be specific, 2) have timely electronic data entry, 3) integrate multiple laboratories using uniform protocols and databases, 4) be used by the agencies responsible for disease control, and 5) implement statistical methods for detecting departures from background levels in both time and space. We have detected clusters of shigellosis of public health importance, which have been confirmed by PFGE as consisting of closely related clones, and informed local public health efforts. This WHONET-SaTScan system of data organization and analysis could represent a good complementary tool for national surveillance system, for early outbreak detection in real time, signalling the importance to investigate some events , and could be applied to other pathogens, implemented by other networks of laboratories, and scaled up to national and international levels.
